# Delayed immune-related events (DIRE) after discontinuation of immunotherapy: diagnostic hazard of autoimmunity at a distance

**DOI:** 10.1186/s40425-019-0645-6

**Published:** 2019-07-03

**Authors:** Marcus A. Couey, R. Bryan Bell, Ashish A. Patel, Meghan C. Romba, Marka R. Crittenden, Brendan D. Curti, Walter J. Urba, Rom S. Leidner

**Affiliations:** 1 Robert W. Franz Cancer Center, Providence Portland Medical Center, 2N35 North Pavilion, 4805 N.E. Glisan St, Portland, OR 97213 USA; 2 Providence Neurological Specialties-West, Providence St. Vincent Medical Center, 9135 Southwest Barnes Road, Suite 461, Portland, OR 97225 USA; 3 The Oregon Clinic, Radiation Oncology, 4805 NE Glisan St. Garden Level, Portland, OR 97213 USA

**Keywords:** Immunotherapy, Checkpoint inhibitor, Costimulatory agonist, Delayed toxicity, Immune-related adverse events

## Abstract

**Background:**

The risk of delayed autoimmunity occurring months or years after discontinuation of immunotherapy is frequently asserted in the literature. However, specific cases were rarely described until 2018, when a wave of reports surfaced. With expanding I-O indications in the adjuvant/neoadjuvant curative setting, growing numbers of patients will receive limited courses of immunotherapy before entering routine surveillance. In this context, under-recognition of DIRE could pose a growing clinical hazard.

**Methods:**

The aim of this study was to characterize DIRE through identification of existing reports of delayed post-treatment irAE in cancer patients treated with immunotherapy. We performed a PubMed literature review from 2008 through 2018 to determine the median data safety reporting window from existing I-O clinical trials, which we then applied to define the DIRE cutoff, and collated all qualifying reports over the same time span. DIRE was defined as new immune-related adverse events (irAE) manifesting ≥90 days after discontinuation of immunotherapy.

**Results:**

Median duration of I-O clinical trials data safety reporting was 90 days (82% ≤ 90 days). DIRE cutoff was thus set as ≥90 days post-immunotherapy. We identified 23 qualifying cases; 21 by literature review and 2 from our institution. Median off-treatment interval to DIRE was 6 months (range: 3 to 28). Median cumulative immunotherapy exposure was 4 doses (range: 3 to 42). Involvement included endocrine, neurologic, GI, pulmonary, cardiac, rheumatologic and dermatologic irAE.

**Conclusions:**

As immunotherapy indications expand into the curative setting, often with brief exposure and potentially sequenced with multimodality treatments, it will be necessary to recognize an emerging diagnostic complex, which we have termed delayed immune-related events (DIRE). Clinical vigilance has the potential to reduce morbidity from diagnostic delay, as irAE are generally manageable with prompt initiation of treatment – or from misdiagnosis - as misattribution can lead to unnecessary or harmful interventions as we describe. DIRE should therefore figure prominently in the differential diagnosis of patients presenting with illnesses of unclear etiology, irrespective of intervening treatments or interval post-immunotherapy, both of which can confound diagnosis. Increased recognition will rest on delineation of DIRE as a clinical diagnostic entity.

**Electronic supplementary material:**

The online version of this article (10.1186/s40425-019-0645-6) contains supplementary material, which is available to authorized users.

## Background

Two recent cases of severe autoimmune illness in patients treated by our institution’s Head and Neck Oncology Department were the impetus for this study (Additional file 1: Institutional Case Series). Both cases were diagnosed months to years after brief exposure to immunotherapy in previous neoadjuvant surgical window trials. In Case No. 1, the relapsing remitting course of neurologic deficits roughly two years after a one week course of immunotherapy (anti-OX40 agonist mAb), led to diagnostic disputes among consulting specialists and resulted in the placement of an Ommaya reservoir based on an erroneous diagnosis of leptomeningeal carcinomatosis, ultimately requiring transfer through three hospital systems to arrive at a conclusive diagnosis of neurosarcoidosis. We found a prevailing assertion in the literature that new autoimmune illness, presenting months to years after discontinuation of immunotherapy, has been well-described. And yet, on tracing the citation trail from 39 review articles, we were able to identify only a single case report (cited by several reviews), describing a patient with delayed dermatologic hypersensitivity reaction (swelling and >erythema of the upper extremities with pruritus) diagnosed 76 days after the last dose of ipilimumab [1]. This prompted us to ask, to what extent are post-immunotherapy immune-related adverse events (irAE) being reported in the literature, what barriers exist to recognition, and to what extent have the hazards of misdiagnosis been described.

## Methods

The aim of this study was to characterize DIRE through identification of existing reports of delayed post-treatment irAE in cancer patients treated with immunotherapy. Literature review was conducted in two phases. In the first phase, we queried existing clinical trials to determine a temporal cutoff by which to define DIRE. In the second phase, we applied this cutoff to identify relevant case reports over the same period, and included 2 cases from our institution.

### Defining temporal cutoff for DIRE

PubMed search of English language reports from Jan 1, 2008 to Dec 31, 2018 was conducted using first-pass Boolean filtering followed by automated titles keyword filtering (Additional file 2: Supplementary Methods). Entries were then divided into two groups: 1) I-O clinical trials, or 2) irAE or toxicity-related case reports. The text, supplement and on-line protocol (when available) for each confirmed I-O clinical trial was manually reviewed to identify the duration of serious adverse event (SAE) reporting. The median SAE reporting duration, thus determined, was taken as the temporal cutoff to define DIRE.

### Literature search for clinical cases

We next performed full text keyword search of all identified entries (trials and case reports), using: “after,” “delay,” “time,” “onset,” “days,” “week,” and “month.” Articles that described irAE after immunotherapy discontinuation were manually reviewed, including recursive bibliography search for any additional reports. Identified DIRE cases were confirmed by consensus reading of at least two investigators. Cases in which an irAE had been previously diagnosed (e.g. colitis first noted on-treatment, then recurring post-treatment), were considered recrudescent and excluded.

## Results

### Determining a temporal cutoff for DIRE (Fig. 1)

**Fig. 1:**
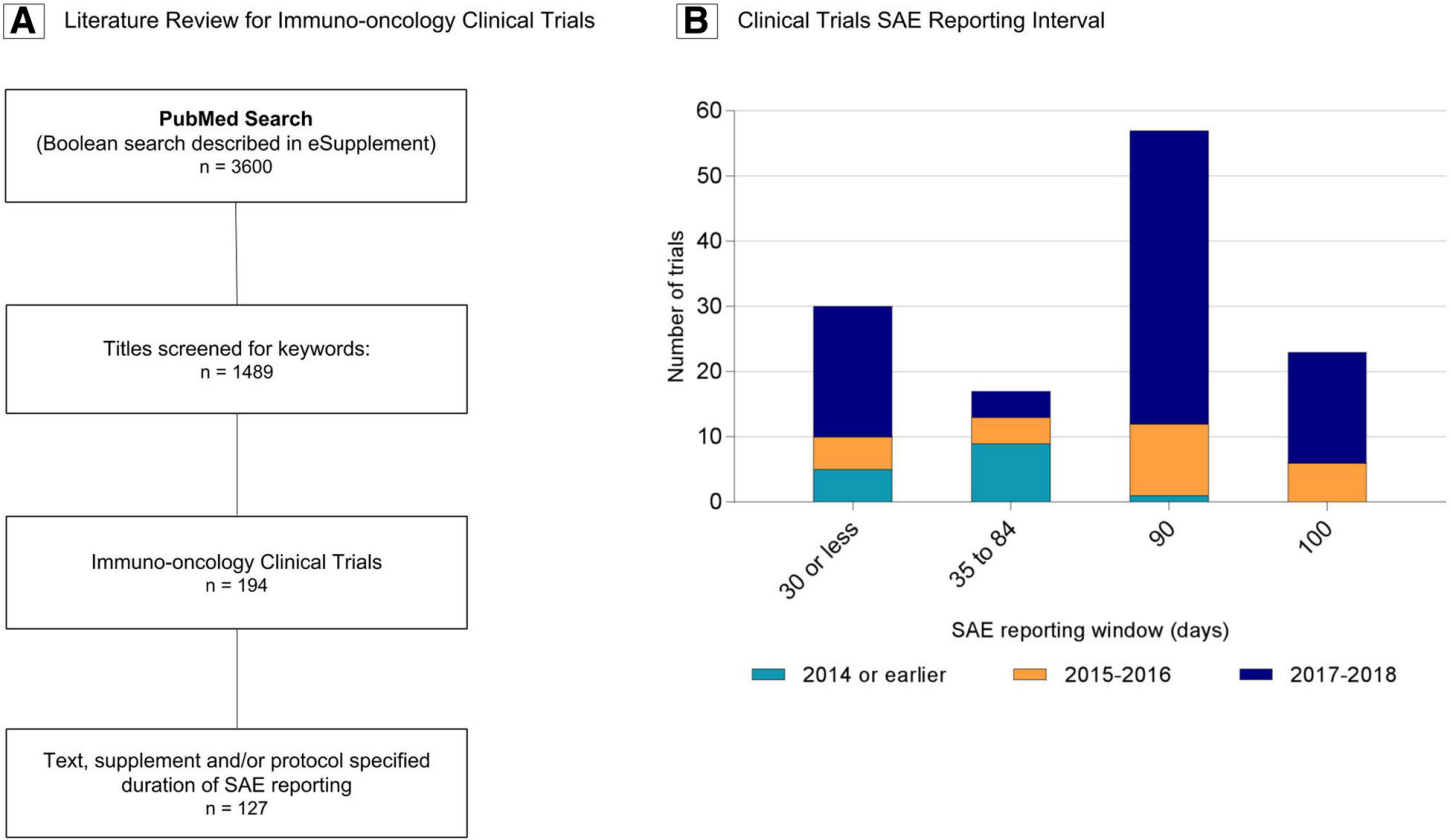
Serious adverse event (SAE) reporting. **a** Literature review to identify immuno-oncology clinical trials. **b** Clinical trials SAE reporting duration after discontinuation of immunotherapy

PubMed Boolean filter and sequential automated titles keyword filter resulted in 3600 and 1489 entries, respectively (Fig. 1a). Of these, 194 were I-O clinical trials, of which 127 specified a discrete duration of SAE reporting in the report or via on-line protocol (Fig. 1b). The duration of SAE reporting following the last dose of immunotherapy was 90 days or less in 82% of I-O clinical trials (range 28 to 100 days). Extension of the SAE reporting window to 90 or 100 days appears to be a more recent trend, accounting for only 7% (1/15) of pre-2015 trials identified. The median I-O trials SAE reporting window (90 days), thus determined, was taken as the temporal cutoff to define DIRE cases the literature search which followed.

### Literature search for DIRE cases (Fig. 2)

**Fig. 2:**
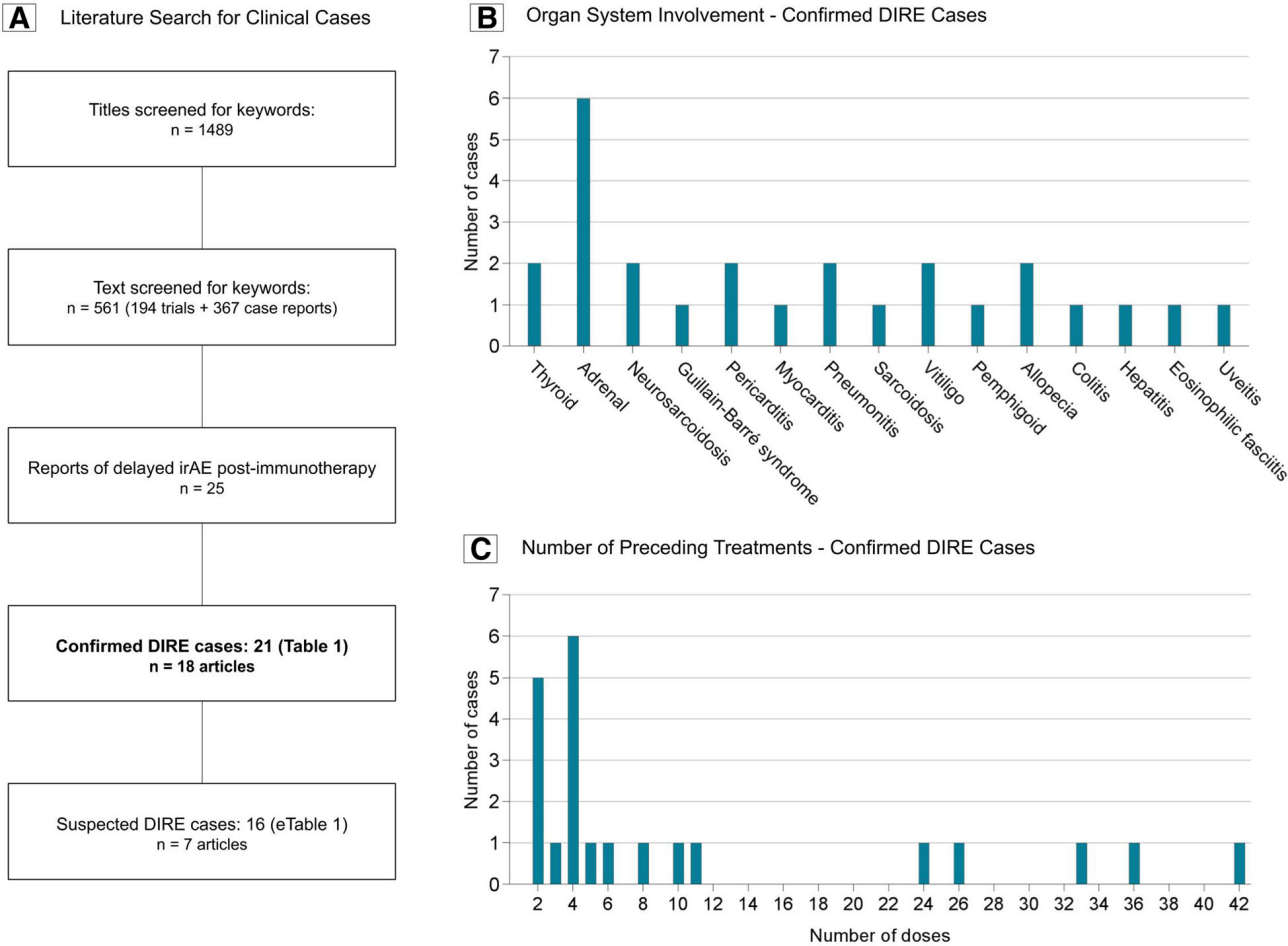
DIRE cases. **a** Literature review to identify DIRE cases – immune-related adverse events presenting ≥90 days after discontinuation of immunotherapy

Literature search identified 367 case reports, case series and review articles in which the title suggested relevance to irAE or I-O toxicity. These were combined with the 194 I-O clinical trials identified by preceding search, for full-text keyword screening of *n* = 561 reports (Fig. 2a). We identified 21 cases of DIRE using a definition of autoimmune sequelae newly diagnosed ≥90 days following discontinuation of immunotherapy [2–19]. Only one case could be confirmed from an I-O trial, reflecting the lack of granularity inherent to conventional SAE reporting (eg. timing of SAE onset relative to first or last treatment dose) [15]. Notably, over half the cases came from 2018 (11 of 21). Two local cases are described in this report, bringing the total to 23 DIRE cases (Table 1). Literature search additionally identified 16 suspected DIRE cases, primarily from I-O clinical trials (14/16), which could not be confirmed or excluded due to lack of sufficient detail and are included in the online supplement (Additional file 3: Table S1).

**Table 1:** DIRE Cases

Case	Disease / age / sex	Rx Setting	DIRE	Post IO (months)	Drug	Doses (total)	Best Response	Notes
1	HNSCC, HPV+ / 62 / M	Neoadjuvant	Neurosarcoidosis	28^a^	MEDI6469 (OX40 agonist)	3	N/A	Couey et al., 2019; Interim treatments: Surgical resection and adjuvant RT (2Gy × 33)
2	HNSCC, HPV+ / 62 / M	Neoadjuvant	Adrenal Insufficiency; Encephalopathy, acute	4^a^	Nivolumab	2	N/A	Couey et al., 2019; Interim treatments: Surgical resection and adjuvant RT (2Gy × 27)
3	Melanoma / 64 / F	Adjuvant	Pneumonitis	8	Nivolumab or Ipilimumab (remains blinded)	7 or 4 (blinded)	NED at 2 y	Mandalà et al., 2018 [2]; On-treatment irAE: Colitis; Interim treatments: Infliximab, Corticosteroids
4	Melanoma / 62 / F	Adjuvant	Pneumonitis	6	Nivolumab	5^b^	Brain met at 4 mo	Diamantopoulos et al., 2017 [3]; On-treatment irAE: Thyroiditis, Hepatitis; Interim treatments: Methimazole
5	Melanoma / 55 / M	Adjuvant	Hypothyroidism	3	Ipilimumab	2	NED at 4 mo	Garcia et al., 2018 [4]; On-treatment irAE: AIDP, Adrenal insufficiency; Interim treatments: Corticosteroids
6	Melanoma / 63 / M	Metastatic	Colitis	23	Pembrolizumab	33^b^	Not reported	Sarofim and Winn, 2018 [5]; Underwent hemicolectomy for pseudo-obstruction, final path ICI-induced colitis
7	NSCLC / 60 / M	Metastatic	Adrenal Insufficiency	15	Pembrolizumab	24	CR	Boudjemaa et al., 2018 [6];
8	Melanoma / 65 / M	Metastatic	Neurosarcoidosis	11	Ipilimumab + Nivolumab	2	SD	Tan et al., 2018 [7]; On-treatment irAE: Colitis, Transaminitis; Pulmonary sarcoidosis 1 month post-IO; interim treatments: Infliximab, Corticosteroids
9	Melanoma / 70 / M	Metastatic	Vitiligo	9	Pembrolizumab	4	PD	Hanrahan et al., 2013 [8]; Interim treatments: Corticosteroids for polymyalgia rheumatica 5 months post-IO
10	Melanoma / 77 / F	Metastatic	Hepatitis	8	Ipilimumab followed by Nivolumab	4 (Ipi)^b^ 22 (Nivo)^b^	PR	Parakh et al., 2018 [9]; Interim treatments: RT 6Gy × 6 (adrenal met)
11	NSCLC / 73 / M	Metastatic	Adrenal Insufficiency	7	Nivolumab	4	SD	Shrotriya et al., 2018 [10]; Concurrent treatments: Gemcitabine/Vinorelbine
12	NSCLC / 66 / M	Metastatic	Adrenal Insufficiency	6	Nivolumab	11	PD	Otsubo et al., 2018 [11];
13	SCC cutaneous / 80’s / F	Metastatic	Bullous Pemphigoid	6	Pembrolizumab	4	PD	Wang et al., 2018 [12]; On-treatment irAE: Erythema multiforme
14	Melanoma / 67 / F	Metastatic	Alopecia	6	PD-1 + CTLA4	2	Not reported	Zarbo et al., 2017 [13]; On-treatment irAE: Colitis; Interim treatments: Infliximab, Corticosteroids
15	Melanoma / 65 / F	Metastatic	Pericarditis	6	Ipilimumab	4	CR	Dasanu et al., 2017 [14]; On-treatment irAE: Thyroiditis, Transaminitis, Rash; Inflamm. arthritis at 2mo & 8mo); Interim treatments: Corticosteroids
16	“Melanoma or solid tumor”^c^	Metastatic	Uveitis	5	Tremelimumab + PF-3512676 (TLR9 agonist)	4 (Treme) 38 (TLR9)^b^	Not reported	Millward et al., 2013 [15]; On-treatment irAE: Neutropenia (TLR9); Rectal bleeding (Treme)
17	Melanoma / 60’s / M	Metastatic	Sarcoidosis (pulmonary, cutaneous)	5	Pembrolizumab	10	CR	Wang et al., 2018 [12];
18	NSCLC / 68 / F	Recurrent	Adrenal Insufficiency	4	Nivolumab	2	PR	Otsubo et al., 2018 [11]; On-treatment irAE: Pneumonitis; Interim treatments: Corticosteroids
19	Melanoma / 63 / F	Metastatic	Myocarditis	4	Ipilimumab	8	SD	Roth et al., 2016 [16]; On-treatment irAE: Hypophysitis, Adrenal insufficiency; Interim treatments: Surgery (recurrence), Corticosteroids
20	Melanoma 65 / F	Metastatic	Alopecia	3	PD-1 + CTLA4	4	PR	Zarbo et al., 2017 [13]; On-treatment irAE: Transaminitis
21	Melanoma / 63 / F	Metastatic	Eosinophilic Fasciitis; Encephalopathy, acute	3	Pembrolizumab	36^b^	CR	Khoja et al., 2016 [17]; Myalgia 1 month post-IO
22	Melanoma / 81 / F	Metastatic	Guillain–Barré Syndrome	3	Pembrolizumab	6	PD	Khoja et al., 2015 [18]; On-treatment irAE: Thyroiditis; Rash 7d after subsequent BRAF inhibitor, 1mo post-IO; Interim treatments: Corticosteroids
23	Melanoma / 59 / M	Metastatic	Adrenal Insufficiency; Pericarditis; Hypothyroidism	3	Ipilimumab	4	Not reported	Yun et al., 2015 [19];

Abbreviations: *IO* Immuno-oncology, *HNSCC* Head and neck squamous cell carcinoma, *NSCLC* Non-small cell lung cancer, *RT* Radiotherapy, *ICI* Immune-checkpoint inhibitor, *AIDP* Acute inflammatory demyelinating polyneuropathy, *Nivo* Nivolumab, *Ipi* Ipilimumab, *Treme* Tremelimumab, *NED* No evidence of disease, *mo* Months, *y* Years, *CR* Complete response, *PR* partial response, *SD* Stable disease, *PD* Progressive disease

a Cases from our institution

b Estimate from article narrative

c Tumor type was not specified, unkown age / sex

### Characteristics of DIRE cases (Table 1)

The median interval to DIRE diagnosis was 6 months post-immunotherapy (range 3 to 28 months). Target organ system involvement (Fig. 2b) included: endocrine (7 patients), cutaneous (5), neurologic (5), pulmonary (3), cardiac (3), gastrointestinal (2), rheumatologic (1) and ophthalmologic (1). Interestingly, over half of the DIRE cases occurred following a brief immunotherapy course of ≤4 doses (Fig. 2c: median 4 doses, range 3 to 42). Immunotherapy exposure included pembrolizumab, nivolumab, ipilimumab, tremelimumab plus PF-3512676 (TLR9 agonist) and MEDI6469 (anti-OX40 mAb agonist). In 5 cases, patients received both anti-PD1 and anti-CTLA-4, sequentially or concurrently. Three patients experienced 2 or more DIRE, while a distinctly separate group of patients, roughly 50% (12/23), experienced prior on-treatment irAEs - frequently the proximal cause of immunotherapy discontinuation (9/12) - of which most were managed with corticosteroids (7/12), with the addition of infliximab for cases of colitis (3/12).

Melanoma predominated (15 cases), followed by non-small cell lung cancer (NSCLC) (4), HPV-associated head and neck squamous cell carcinoma (HNSCC) (2), cutaneous squamous cell carcinoma (1) and not specified (1; likely melanoma, clinical trial) [15]. The majority of DIRE cases occurred in the recurrent/metastatic setting (18/23), but three cases were reported following adjuvant melanoma treatment; we identified two cases following neoadjuvant surgical window I-O trials in HNSCC. Subsequent treatment following immunotherapy included: BRAF inhibitor for a melanoma patient (to which Guillain-Barré syndrome was erroneously attributed), and multimodality standard of care (surgery followed by risk-adapted radiation +/− chemotherapy) for the two HNSCC patients, both of which developed protracted neurologic DIRE, with adrenal crisis included in the DIRE complex in case No. 2 (Table 1; Additional file 1: Institutional Case Series).

### Suspected DIRE cases – incomplete reports

There were 16 additional suspected DIRE cases identified by literature search, which although likely representing bona fide examples, could not be included due to insufficient reported details [20–26]. (Additional file 4: Clinical Trial AEs Suspicious for DIRE) Target organ system involvement included endocrine (6 patients), cutaneous (6), gastrointestinal (3), genitourinary (1), hematologic (1), rheumatologic (1) and ophthalmologic (1). Immunotherapy exposure included pembrolizumab, nivolumab, ipilimumab and gp100. Melanoma predominated (15 cases) of which 20% were adjuvant (3/15); the sixteenth case was metastatic NSCLC.

## Discussion

The variable temporality of *on-treatment* irAE is well-characterized [27–37]. However, o*ff-treatment autoimmune illness,* manifesting months to years after discontinuation of immunotherapy, have occasionally been alluded to in the literature, but probably represent an under-recognized phenomenon [38–42]. In 2013, the first such report involving a case of vitiligo manifesting 9 months after discontinuation of pembrolizumab appeared in abstract form in *Deutschen Dermatologischen Gesellschaft* (case No. 9, Table 1) [8]. Through 2017, a handful of reports described cases of post-immunotherapy autoimmune illness, but focused primarily on target organ manifestations, rather than the fact of delayed onset following an extended interval off-treatment and the diagnostic challenges this most likely posed [3, 13, 16–19]. By the close of 2018, however, a wave of new reports had surfaced, some explicitly emphasizing the significance of newly manifest irAE emerging long after discontinuation of immunotherapy, a clinical diagnostic complex we have termed DIRE (delayed immune-related events).

To our knowledge, this is the first study to collate existing scattered reports of substantially delayed irAE, manifesting at prolonged intervals after discontinuation of immunotherapy. Over half of all cases occurred following short courses of immunotherapy of ≤4 doses, and the majority of cases were reported in 2018 (Table 1). There is sound mechanistic plausibility to support these observations. For instance, PD-1 receptor occupancy on T cells plateaus at approximately 80% up to 90 days after a single dose of nivolumab, despite a serum half-life of only 12–20 days. After three doses, occupancy remains at 40% for more than 8 months after the last dose [43]. Just as durable clinical responses following immunotherapy appear to be independent of dose and duration of treatment, so too, immune-mediated toxicity would be expected to display variable onset relative to treatment.

Obstacles to assessment of the true frequency and extent of DIRE are inherent in current I-O clinical trials reporting convention. Issues such as limited follow up periods and incompleteness of irAE reporting were previously described by Chen et al. in a systematic review of I-O clinical trials [44]. Some trial protocols explicitly excluded reporting of adverse events occurring > 30 days after last dose, or reporting of any adverse events after initiation of another cancer treatment [45–49]. Importantly, irAE are routinely reported relative to the start of treatment, without any related information regarding the end of treatment [21, 50–54]. We were only able to confirm a single case of DIRE in an I-O clinical trial, despite rather simple criteria we proposed, and a careful manual review of all published trials reports. This was a case of grade 2 uveitis occurring 157 days after end of study treatment (TLR9 + tremelimumab), which was couched in a DLT table that caught our eye on *manual reading of the report* (case No. 16, Table 1), and actually not within the searchable text [15].

The fact that our literature search was able to identify cases dating back to 2013 suggests that DIRE is not a new phenomenon. Indeed, we identified 39 review articles that posited the existence of delayed irAE post discontinuation of treatment [29, 31, 34, 38–42, 44, 55–84]; however, only two of these reviews provided specific citations, both to the same report describing a case of delayed dermatologic hypersensitivity reaction starting 76 days following final dose of ipilimumab [34, 66]. This particular example would not have posed a substantial diagnostic challenge, nor qualified as DIRE based on a ≥ 90 day cutoff. It may be that clinical anecdotes were in circulation as a basis for the widespread assertions we encountered in the literature, but the lack of citations to any extant reports suggest an under-reporting effect, likely deriving from clinical reluctance to recognize autoimmunity at a distance, in favor of misattribution to more proximal events or treatments.

Two recent cases of severe and substantially delayed post-treatment irAE occurring in patients treated by our institution’s Head and Neck Oncology Department are described in this report. These serve as among the first detailed examples of DIRE following *neoadjuvant* immunotherapy, and describe the diagnostic uncertainty posed by otherwise healthy individuals being followed for surveillance, months to years following remote (and brief) immunotherapy exposure in the neoadjuvant clinical trials context. We were unable to find analogous reports in the literature, indicating a diagnostic hazard which is predicted to increase with new I-O approvals in the curative setting (adjuvant/neoadjuvant treatment) [21, 85–92]. Current diagnostic challenges are reminiscent of early experience in allogeneic bone marrow transplantation, prior to delineation of graft versus host disease (GVHD) as a clinical diagnostic complex. Diagnostic misattribution to the effects of intercurrent chemotherapy, radiation, disease recurrence or sepsis, were reported as common confounding factors [93, 94]. Clinical awareness, therefore has the potential to avert morbidity/mortality from misdiagnosis, by preventing unnecessary and potentially harmful interventions, (as in the neurosarcoidosis case we describe of Ommaya reservoir placement on the basis of an erroneous leptomeningeal carcinomatosis diagnosis) – and from diagnostic delay, given that these conditions are generally manageable with prompt initiation of immune suppressive therapy.

As contemporary examples of diagnostic hazard, our literature search identified two reports, one of immune-related colitis manifesting 23 months after completion of pembrolizumab, in which the patient was initially treated with neostigmine based on an erroneous diagnosis of Ogilvie’s syndrome, failed to respond, and then underwent right-sided colectomy, which unfortunately revealed correct diagnosis (Case No. 6, Table 1) [5]. A case of Guillain-Barré syndrome (GBS) manifesting 3 months post-pembrolizumab, was erroneously attributed to subsequent treatment with dabrafenib (case No. 22, Table 1) [18]. Only one other case of GBS has been associated with dabrafenib, in a 2015 abstract, again post-pembrolizumab [95], whereas the association of checkpoint blockade with GBS is well-recognized [96, 97]. Clinical confounding, akin to experience prior to categorization of GVHD, attests to the need for characterization of DIRE as a clinical diagnostic entity. These cases illustrate several hazards of diagnostic misattribution, including unnecessary/invasive procedures, delay in the proper management of an autoimmune illness, and abandonment of an otherwise beneficial therapy (eg…BRAF inhibitors).

We would highlight three important limitations to this study. First, it is impossible to prove direct causality between immunotherapy and delayed autoimmunity, particularly from case reports. With rare exception, autoimmune sequelae of immunotherapy remain diagnoses-of-exclusion in clinical practice. The lack of a temporal association or dose-dependent relationship, which might otherwise enhance credulity, lends to a cautious skepticism which only further empiric observation will dispel or reinforce. Second, there is a heterogeneous level of detail regarding diagnosis and clinical management in the case reports thus far available. As clinical awareness increases, we anticipate that a growing emphasis will be placed on diagnostic algorithms, management strategies, and possibly unique DIRE associations to specific drugs. Third, we cannot accurately begin to gauge the frequency or true extent of DIRE given clinical trial reporting conventions which favor attribution of irAE to the most temporally proximal treatment/process and reporting irAE relative only to *start* of treatment but not *end* of treatment.

## Conclusions

With expanding I-O indications in the curative disease setting, either as adjuvant/neoadjuvant therapy, or in combination with multimodality approaches, growing numbers of patients will be exposed to immunotherapy before transitioning to routine clinical surveillance. In this context, it will be necessary to recognize an emerging clinical diagnostic complex, which we have termed DIRE (delayed immune-related events), manifesting months to years after discontinuation of immunotherapy and representing a substantive diagnostic hazard. In our experience, diagnostic misattribution is clinically reinforced by several confounding factors: 1) a brief and remote immunotherapy exposure; 2) intervening treatments with overlapping toxicities; 3) a protracted diagnosis-by-exclusion process; 4) reduced vigilance in the post-treatment setting. DIRE syndrome should therefore figure prominently in the differential diagnosis of patients presenting with unexplained illnesses, irrespective of post-immunotherapy interval. The clinical hazards of diagnostic misattribution include inappropriate invasive procedures (unnecessary colectomy or Ommaya reservoir placement, as in cases described) and delay to start of treatment for autoimmune conditions that are often readily managed with prompt initiation of immune suppressive therapy. Changes to I-O clinical trial reporting convention could unmask the true incidence of DIRE, and include: the standardization of irAE reporting duration; collection of safety data regardless of whether a new cancer therapy is started; publication of irAE that emerge after the formal study period; and reporting of time-to-onset of irAE in relation to both treatment start *and* end.

## Additional files

Additional file 1: Institutional Case Series. Detailed descriptions of two DIRE cases identified from our institution. (DOCX 23 kb)

Additional file 2: Supplementary Methods. Boolean search strategy and keywords for titles screen (DOCX 17 kb)

Additional file 3: **Table S1.** Table of suspected DIRE cases that could not be confirmed due to lack of supporting information (PDF 23 kb)

Additional file 4: Clinical Trial AEs Suspicious for DIRE. Descriptions of suspected DIRE cases from clinical trials that could not be confirmed due to lack of supporting information (DOCX 18 kb)

## Data Availability

The data and references supporting the conclusions of this article are included within the main text and additional files.

## Abbreviations

DIRE: Delayed-immune related events
DLT: Dose-limiting toxicity
GBS: Guillain-Barré syndrome
GVHD: Graft versus host disease
HNSCC: Head and neck squamous cell carcinoma
I-O: Immuno-oncology
irAE: Immune-related adverse event
mAb: Monoclonal antibody
NSCLC: Non-small cell lung cancer
SAE: Serious adverse event
