# Supplementary material for: Delayed immune-related events (DIRE) after discontinuation of immunotherapy: diagnostic hazard of autoimmunity at a distance
Source: J Immunother Cancer. 2019 Jul 3;7:165. doi: 10.1186/s40425-019-0645-6 (PMC6609357; doi:10.1186/s40425-019-0645-6)
Supplement: Supplementary file 1 — Institutional Case Series. Detailed descriptions of two DIRE cases identified from our institution. (DOCX 23 kb) [file 40425_2019_645_MOESM1_ESM.docx]

**Institutional Case Series:**

*Case No. 1: Neurosarcoidosis*

A 62-year-old otherwise healthy male with HPV-associated HNSCC of the right tonsil (cT1 cN2 M0, AJCC 7^th^ ed.) was treated in January 2016 via clinical trial of neoadjuvant MEDI6469 OX40 agonist murine monoclonal antibody, 0.4 mg/kg IV x3 over one week M-W-F (NCT02274155), which was then followed by robotic oropharyngectomy and right neck dissection (ypT1 ypN3 M0) two days after the third/final dose of MEDI6469. This was followed by risk-adapted adjuvant 66 Gy IMRT, complicated at the outset by a one week hospitalization in March 2016 for fever with leukocytosis, acute mental status changes (hallucinations), myoclonus and meningismus, following prophylactic feeding tube placement. Evaluation at that time included: MRI brain/neck showing mild ventricular enlargement with fluid levels layering in the lateral horns and anterior cortical destruction of the C2-C3 vertebral bodies; lumbar puncture (opening pressure of 23 mmHg) with CSF indices showing a neutrophil predominant pleocytosis, low glucose, elevated protein; and EEG which showed pattern consistent with moderate non-specific encephalopathy sans epileptiform activity. Neurology consultation recommended treatment for presumed bacterial meningitis and vertebral seeding, with empiric IV antibiotic coverage and dexamethasone 10mg IV q6h for four days. Initiation of steroids resulted in dramatic resolution of clinical signs & symptoms within 48 hours and the patient was discharged home to complete a 6-week course of IV ceftriaxone. However, bacterial and fungal cultures from both blood and CSF did not ultimately identify an infectious etiology, nor did ancillary CSF testing, which included AFB & India ink stains, HSV/Enterovirus PCR, and cytology which showed abundant acute inflammatory cells, sans malignant cells. He was able to subsequently complete adjuvant radiation by April 2016, without further complications. In October 2016, he was seen by his surgeon with a negative surveillance PET/CT, at which point his feeding tube was removed; he then relocated to a remote rural region near the Idaho border and was lost to follow-up.

In May 2017 he was evaluated in a local ER for two episodes of expressive aphasia lasting an hour at a time, diagnosed as TIA following negative CT head and carotid US doppler. Starting in January 2018 he began to experience relapsing/remitting episodes of inferior visual field deficits and ataxia leading to the use of a walker. He underwent thorough outpatient evaluation by his local physicians, including a neurologist starting in March 2018, with testing that included CT head + CT angio, MRI brain, repeat carotid US doppler, EEG, Holter monitor and cardiac echo – all of which failed to identify a significant pathology to account for his progressive functional decline, until May of 2018 when he was hospitalized via his local ER following repeat CT head which now revealed a new communicating hydrocephalus. This prompted transfer for increased level of care to an adjacent out-of-state hospital, where neurology consultation attributed his roughly 12 month course of gradual clinical decline to leptomeningeal carcinomatosis (LMC), based on a repeat MRI series which showed somewhat equivocal nodular enhancement (original negative formal read from radiologist was addended). The diagnosis of LMC persisted, despite lumbar puncture x2 negative for malignant cells on cytology (opening pressure reported as normal; CSF indices showing low glucose, elevated protein and mild lymphocytic pleocytosis; CSF flow cytometry demonstrating polycolonal B cells). An Ommaya reservoir was next placed by neurosurgery, at which time dural and R frontal lobe biopsies were also obtained, which were negative for neoplasia, inflammation or necrosis. An impasse then arose between consultants, as the consulting oncologist demurred to treat in the absence of cytologic evidence of LMC, and the patient was subsequently transferred by the consulting neurologist to our center by fixed-wing medical transport. The presumed diagnosis of LMC was persistently invoked by multiple consulting specialists at our center, the exception being medical and radiation oncology, who argued that untreated LMC would not be survivable for a year nor be characterized by a smoldering relapsing/remitting course. Repeat MRI spine showed patchy dural enhancement from cervical to lumbar regions, absent nodularity. Multiple repeat CSF analyses were performed (Ommaya x3 and LP x1), which were consistently negative for malignant cells (flow cytometry showing 67% CD4 and 22% CD8 T cell predominance). Additional negative diagnostic testing included: HIV and MTB screen, paraneoplastic antibody panel, GAD Ab, IgG index/SPEP, oligoclonal bands, VDRL, JCV, HSV, VZV, B12, MMA, enterovirus, coccidioides Ab, cryptococcus Ag, histoplasma Ag, fungal and routine cultures, ANA panel/dsDNA/ANCA panel/RF/CRP/Thyroid peroxidase Ab, HPA endocrine axis screen, autoimmune encephalopathy panel, AQP-4-IgG and MOG-IgG1. *The sole positive finding, was a CSF ACE level = 7.6 (0.0 to 2.5 U/L) on repeat LP, consistent with neurosarcoidosis – late May 2018.* After nearly two weeks of specialized evaluation and testing, the consulting neurology team initiated a trial of pulse methylprednisolone 500mg IV q12h, which led to abrupt overnight improvement in aphasia, ataxia and other smoldering deficits, aside from subtle inferior visual field cuts. Three days later, he was discharged on oral prednisone 60 mg daily in early June 2018. He is being followed in the neuroimmunology clinic for neurosarcoidosis, for which he is maintained on oral prednisone with ongoing slow taper as of January 2019. Functionally, he no longer requires a walker and lives at home, but appears to have irreversible inferior visual field deficits bilaterally, with neuro-ophthalmologic exam showing superior optic disc pallor and optical coherence tomography showing superior RNFL thinning, thought to suggest sequelae of bilateral optic neuropathy.

*Case No. 2: Adrenal Insufficiency and Acute Encephalopathy*

A 62-year-old male with HPV-associated HNSCC of the right tonsil (cT1 cN2b M0, AJCC 7^th^ ed.) was treated in November 2017 via clinical trial of neoadjuvant nivolumab 240mg IV days 1 and 15 (NCT02488759) followed by day 27 restaging CT neck showing PD by RECIST 1.1 (24% increase in target lesions) and planned day 34 robotic oropharyngectomy and right neck dissection (ypT1 ypN2 M0, AJCC 8^th^ ed). Risk-adapted adjuvant 60 Gy IMRT started within 6 weeks of surgery in January 2018, but was cut short at 54 Gy, due to hospitalization for grade 4 oral mucositis requiring feeding gastrostomy tube placement in February 2018, complicated by transient refeeding syndrome. After two days at home in March 2018, he was readmitted via the ED for altered mental status and acute DVT of bilateral lower extremities. The ensuing one month hospital course was complex, characterized by a stepwise physiologic decompensation, beginning with hemodynamic instability and hypoxemia (misattributed to aspiration pneumonia), which fairly quickly devolved to ICU transfer on hospital day 3 for vasopressor support, and culminated in intubation on hospital day 12 following a relapsing/remitting course of encephalopathy with Cheyne-Stokes breathing pattern.

Clinical misattribution to sepsis and delirium persisted, absent evidence of infectious source or contributing etiology from exhaustive neurology evaluation which included MRI imaging, lumbar puncture and continuous EEG monitoring. Fortunately, on hospital day 12 at the time of intubation, a serum cortisol level was sent which returned non-detectable (< 1 ug/dL; tandem ACTH not obtained). Stress-dose hydrocortisone 50mg IV q6h was initiated based on this result - he was weaned from vasopressors by hospital day 14, extubated by hospital day 15, transferred out of the ICU by hospital day 17, and delirium fully resolved by hospital day 22. He was maintained on oral adrenal replacement (po hydrocortisone 20mg/10mg) and returned home in April 2018 after one week of intensive inpatient rehabilitation. Outpatient endocrine evaluation included: repeat MRI pituitary (negative for hypophysitis); cosyntropin 250mcg IV stimulation test (serum ACTH < 5 pg/mL; 0-min cortisol = 1.0 ug/dL, 30-min cortisol = 3.6 ug/dL, 60-min cortisol = 4.8 ug/dL); TSH = 6.94 mIU/L, free T4 = 1.1 ng/dL; Testosterone total = 393 ng/dL, free = 42 pg/mL; IGF-1 = 245 ng/mL; LH = 4.5 mIU/mL and FSH = 14.8 mIU/mL. He has since returned to working full-time, but as of January 2019, has not yet been able to wean from adrenal replacement therapy, despite several tapering attempts.
