# Supplementary material for: Delayed immune-related events (DIRE) after discontinuation of immunotherapy: diagnostic hazard of autoimmunity at a distance
Source: J Immunother Cancer. 2019 Jul 3;7:165. doi: 10.1186/s40425-019-0645-6 (PMC6609357; doi:10.1186/s40425-019-0645-6)
Supplement: Supplementary file 2 — Supplementary Methods. Boolean search strategy and keywords for titles screen (DOCX 17 kb) [file 40425_2019_645_MOESM2_ESM.docx]

**Supplementary Methods**

*Boolean Search:*

PubMed was accessed and queried on January 7, 2019. Boolean search terms were: (checkpoint OR costimula* OR co-stimula* OR nivolumab OR pembrolizumab OR avelumab OR atezolizumab OR PD-1 OR PD1 OR durvalumab OR PD-L1 OR PDL1 OR ipilimumab OR tremelimumab OR CTLA-4 OR CTLA4 OR OX40 OR 4-1BB OR GITR

OR CD27 OR CD40 OR ICOS) AND (adverse event* OR adverse reaction OR toxicit* OR irAE OR neurological OR hypophysitis OR encephalopathy OR meningitis OR thyroid* OR adrenal OR pneumonitis OR colitis OR dermatologic OR cutaneous OR

vitiligo OR myocarditis OR arthritis OR neuropathy OR myopathy OR sarcoidosis OR nephr*) AND (delay* OR late OR timing OR after OR following OR year* OR months OR weeks) NOT allerg* NOT pollut* NOT mice NOT mouse NOT murine NOT rat NOT

rats NOT monkey* NOT pig NOT bovine NOT rabbit NOT fetal NOT neonatal NOT pregnan* NOT miscarriage NOT menopaus* NOT particle* NOT nanoparticle* NOT hydrocarbon NOT vitro[Title] NOT vivo[Title] NOT preclinical[Title] NOT GVHD[Title] ] NOT graft[Title] NOT host[Title] NOT transplan*[Title] NOT reject*[Title] NOT allogeneic[Title] NOT allograft[Title].

*Titles Keyword Screen:*

“checkpoint inhibitor” or “costimulatory agonist” or “immunotherapy” or any recognized immunotherapeutic agent in the context of cancer treatment.
