# Supplementary material for: Delayed immune-related events (DIRE) after discontinuation of immunotherapy: diagnostic hazard of autoimmunity at a distance
Source: J Immunother Cancer. 2019 Jul 3;7:165. doi: 10.1186/s40425-019-0645-6 (PMC6609357; doi:10.1186/s40425-019-0645-6)
Supplement: Supplementary file 3 — Table S1. Table of suspected DIRE cases that could not be confirmed due to lack of supporting information (PDF 23 kb) [file 40425_2019_645_MOESM3_ESM.pdf]

Table S1. Incomplete Reports: Suspected DIRE Cases

Disease	Rx Setting	DIRE	Post IO	Drug	Doses (total)	Notes
Melanoma	Adjuvant	Adrenal Insufficiency; Inflammatory Arthritis	≥ 4 months	Pembrolizumab followed by Ipilimumab	unknown	Luu and Major, 2018;[20] On-treatment irAE: Hepatitis; Interim treatments: Corticosteroids (taper completed 2 weeks prior to adrenal crisis)
Melanoma	Adjuvant	Marrow aplasia	> 100 days[a]	Ipilimumab	≥4	Weber et al, 2017[21]
Melanoma	Adjuvant	Colitis	> 100 days[a]	Ipilimumab	≥4	Weber et al, 2017[21]
Melanoma	Uresectable	Hepatitis	234 days[a]	Ipilimumab + Nivolumab	4 (Ipi);[b] 36 (Nivo)[b]	Wolchok et al, 2017[22]
NSCLC	Metastatic	Graves Orbitopathy	6 months[b]	Nivolumab (+docetaxel)	14[b]	Ricciuti et al, 2017;[23] On-treatment irAE: Pneumonitis; Interim treatments: Corticosteroids
Melanoma	Metastatic	Hypothyroidism	> 90 days	Ipilimumab	4	Yamazaki et al, 2015[24]
Melanoma	Metastatic	Hypopituitarism	> 90 days	Ipilimumab	4	Yamazaki et al, 2015[24]
Melanoma	Metastatic	Vitiligo	> 90 days	Ipilimumab	4	Yamazaki et al, 2015[24]
Melanoma	Metastatic	Hypophysitis	> 70 days	Ipilimumab (+dacarbazine)	6[b]	Robert et al, 2011[25]
Melanoma	Metastatic	Vitiligo	> 70 days	Ipilimumab	≥4	McDermott et al, 2013[26]
Melanoma	Metastatic	Vitiligo	> 70 days	Ipilimumab	≥4	McDermott et al, 2013[26]
Melanoma	Metastatic	Vitiligo	> 70 days	Ipilimumab	≥4	McDermott et al, 2013[26]
Melanoma	Metastatic	Vitiligo; Hypothyroidism	> 70 days	Ipilimumab	≥4	McDermott et al, 2013[26]
Melanoma	Metastatic	Hypogonadism	> 70 days	Ipilimumab + gp100	≥4 (Ipi); ≥4 (gp100)	McDermott et al, 2013[26]
Melanoma	Metastatic	Colitis; Proctitis	> 70 days	Ipilimumab + gp100	≥4 (Ipi); ≥4 (gp100)	McDermott et al, 2013[26]
Melanoma	Metastatic	Vitiligo	> 70 days	gp100	≥4	McDermott et al, 2013[26]

Abbreviations: IO, immuno-oncology; NSCLC, non-small cell lung cancer; Nivo, nivolumab; Ipi, ipilimumab

[a] Refers to timing of treatment-related death; timing of irAE diagnosis was not specified.

[b] Estimate from article narrative.
