# Supplementary material for: Delayed immune-related events (DIRE) after discontinuation of immunotherapy: diagnostic hazard of autoimmunity at a distance
Source: J Immunother Cancer. 2019 Jul 3;7:165. doi: 10.1186/s40425-019-0645-6 (PMC6609357; doi:10.1186/s40425-019-0645-6)
Supplement: Supplementary file 4 — Clinical Trial AEs Suspicious for DIRE. Descriptions of suspected DIRE cases from clinical trials that could not be confirmed due to lack of supporting information (DOCX 18 kb) [file 40425_2019_645_MOESM4_ESM.docx]

**Clinical trial AEs suspicious for DIRE:**

**Wolchok JD, Chiarion-Sileni V, Gonzalez R, et al. Overall Survival with Combined Nivolumab and Ipilimumab in Advanced Melanoma. *N Engl J Med*. 2017;377(14):1345-1356.**

*"Two previously unreported deaths in the nivolumab-plus-ipilimumab group that were considered by the investigator to be related to a study drug were reported more than 100 days after the last dose of study drug. A 72-year-old man with a history of heart disease died on day 589 (433 days after receipt of the last dose) owing to cardiac insufficiency and autoimmune myocarditis, approximately 2 months after receiving a single dose of anti–PD-1 therapy outside the context of the trial, and a 69-year-old woman died on day 735 (234 days after receipt of the last dose) owing to liver necrosis, after she had grade 3 elevations in liver-enzyme levels."*

Summary:

- Possible case of DIRE– hepatic necrosis. However, the date of diagnosis is not provided, only the date of death
  - 69F – death due to hepatic necrosis ~7.8 months after last dose of nivo in nivo+ipi group.
- The other case was not DIRE, as the patient had recently received another dose of anti-PD-1
  - 72M – death due to autoimmune myocarditis 2 months after retreatment with PD-1 outside of the original trial.

**Weber J, Mandala M, Del Vecchio M, et al. Adjuvant Nivolumab versus Ipilimumab in Resected Stage III or IV Melanoma. *N Engl J Med*. 2017;377(19):1824-1835.**

*"There were 2 deaths (0.4%) from toxic effects (marrow aplasia and colitis, both of which occurred more than 100 days after the last dose) in the ipilimumab group and no treatment-related deaths in the nivolumab group."*

Summary:

- 2 possible cases of DIRE
  - However, the date of diagnosis is not provided. Only states that death occurred >100 days post IO.

**Yamazaki, N., et al. "Phase II study of ipilimumab monotherapy in Japanese patients with advanced melanoma." *Cancer chemotherapy and pharmacology* 76.5 (2015): 997-1004.**

*“Adverse events that occurred more than 90 days after the last dose of ipilimumab included 2 endocrine irAEs [grade 1 hypothyroidism and grade 2 hypopituitarism (also reported as an SAE)] and 1 skin irAE (grade 1 vitiligo).”*

Summary:

- Three possible cases of DIRE – limited details, no exact timeframe provided (just “> 90 days”)
  - One grade 1 hypothyroidism
  - One grade 2 hypopituitarism
  - One grade 1 vitiligo

**Robert C, Thomas L, Bondarenko I, et al. Ipilimumab plus Dacarbazine for Previously Untreated Metastatic Melanoma. *N Engl J Med*. 2011;364(26):2517-2526**

*“No cases of hypophysitis were noted in the ipilimumab–dacarbazine group except for a single case in a patient receiving maintenance therapy that was reported on day 364 (which was outside the protocol-specified reporting window of <70 days after the last dose — a period representing 5 times the half-life of ipilimumab — and was therefore not categorized as an “on-study” event).”*

Summary:

- Possible case of DIRE – hypophysitis.
  - Does not specify the time elapsed since the last dose, only that it was outside of the 70-day study window.

**McDermott D, Haanen J, Chen T-T, Lorigan P, O’Day S, for the MDX010-20 investigators. Efficacy and safety of ipilimumab in metastatic melanoma patients surviving more than 2 years following treatment in a phase III trial (MDX010-20). *Annals of Oncology*. 2013;24(10):2694-2698.**

*“Among 94 patients who remained alive 2 years after the start of on‐study treatment (54, 24, and 16 patients in the ipilimumab plus gp100, ipilimumab monotherapy, and gp100 monotherapy groups, respectively) 5 patients (9.3%), 1 patient (4.2%), and 1 patient (6.3%), respectively, reported an irAE >70 days after their last dose of study drug. All were grade 1 or 2 in severity (vitiligo, hypothyroidism, diarrhea, proctitis, low blood testosterone, and hypogonadism), with the exception of 1 patient in the ipilimumab plus gp100 group who experienced grade 3 colitis on day 147 that recovered with residual effects.”*

Summary:

- 7 possible cases of DIRE – irAE at least 70 days post IO
  - Doesn't specify the amount of time since the last dose, only that it was >70 days.
